# Interactive Compensation Effects of Physical Activity and Sleep on Mental Health: A Longitudinal Panel Study among Chinese College Students during the COVID-19 Pandemic

**DOI:** 10.3390/ijerph191912323

**Published:** 2022-09-28

**Authors:** Yao Zhang, Jianxiu Liu, Yi Zhang, Limei Ke, Ruidong Liu

**Affiliations:** 1 Soochow College, Soochow University, Suzhou 215006, China; 2 Division of Sports Science & Physical Education, Tsinghua University, Beijing 100084, China; 3 Vanke School of Public Health, Tsinghua University, Beijing 100084, China; 4 Department of Physical Education, Beijing Forestry University, Beijing 100083, China; 5 School of Medicine, Tsinghua University, Beijing 100084, China; 6 Sports Coaching College, Beijing Sport University, Beijing 100084, China

**Keywords:** longitudinal panel study, physical activity, sleep, mental health, interactive compensation effect

## Abstract

Physical activity (PA) and sleep are both important to mental health. However, their joint effects on mental distress have not been well explored. The aim of this study was to investigate the joint effects of PA and sleep on mental health, as well as the dose-response relationships between PA and mental health under different sleep health statuses. A longitudinal panel study was adopted to evaluate the relationship between PA, sleep, and mental health among 66 healthy Chinese college students with four online questionnaire surveys. A mixed-effect model with individual-level random effect was used to analyze the interactive regulation effect of PA and sleep on mental health, and a generalized additive model with splines was further fitted to analyze dose-response relationships between variables. When sleep was at a healthy level, no significant difference in mental health was observed between different levels of PA (*p* > 0.05). However, poor sleepers with moderate and high PA levels indicated significantly fewer negative emotions than those with low PA levels (*p* = 0.001, *p* = 0.004). Likewise, poor sleepers who engaged in more moderate intensity PA could significantly reduce negative emotions (*β* = −0.470, *p* = 0.011) in a near-linear trend. In summary, both sleep and PA benefit mental health, and they probably regulate mental health through an interactive compensation mode. For good and poor sleepers, PA plays a different role in maintaining and improving mental health. Increasing moderate intensity PA up to moderate-and-high levels is recommended for those who simultaneously suffer from sleep and psychological health problems.

## 1. Introduction

Mental health problems undoubtedly have been predominant challenges in contemporary public health, causing long-term impairment throughout life [1]. Converging epidemiological studies indicate that increasing physical activity (PA) is associated with a lower risk of not only chronic diseases, such as cardiovascular, hypertension, and diabetes [2] but also mental disorders [3,4,5]. For people of all age groups, maintaining regular PA protects against mental disorders like stress, anxiety, and depression [6,7]. Promoting PA, even light-intensity PA, may have positive effects on the mental health of older adults [8]. Also, PA improves the quality of life for those who suffer from bipolar disorder, schizophrenia, and posttraumatic stress disorder [9,10]. The beneficial effect of PA on mental health has been proved in animal studies as well. For example, acute exercise increased brain-derived neurotrophic factor (BDNF), an important molecule for synaptic plasticity, thus improving the cerebral structure and helping maintain a relatively stable emotion regulation level [11]. Increasing PA could influence the metabolism via increasing central dopaminergic and noradrenergic activity, enhancing serotonergic activity, and improving mood and mental health [12].

However, some cross-sectional studies reported that increased PA might not predict reduced anxiety and depression, and PA interventions may not be effective enough in promoting mental health [6,13,14]. Moreover, several meta-analyses pointed out that physical activity seemed to have potentially small benefits on mental health, but the evidence was weak, and the association was greatly affected by research methodology as well as individual variations [15,16]. Hence, there were many inconsistencies and even contradictory conclusions on whether and how PA affects mental health. The impact of PA on mental health has become confusing due to differences in age, gender, sleep hygiene, and complex underlying mechanisms.

In addition to PA, sleep also plays an important role in mental health. For instance, sleeping is an important metabolic pathway that clears metabolic waste products (e.g., β-amyloid) in the brain’s interstitial fluid [17] and then directly affects individuals’ mental health [18,19]. Chronic sleep deprivation might lead to pathological anxiety, and the association between sleep and mental health was considered bidirectional because mental disorders could also lead to impairments in sleep [20,21].

Given the important roles of PA and sleep on mental health, recent studies have started to focus on the comprehensive PA-sleep-mental health relationship. Numerous studies have explored the link between PA and sleep and concluded that certain types, appropriate duration, frequency, and intensity of PA could improve sleep quality and duration [22]. A cross-sectional study documented that academic stress directly predicted sleep duration and everyday physical activity duration mediated the relation [23]; a 4-month randomized controlled trial indicated that resistance exercise and stretching led to significant improvements in sleep efficiency for chronic insomniacs and stretching helped to reduce Tension-anxiety [24]. Moreover, one study which pooled two randomized controlled trials indicated that improvements in the overall physical activity and sleep behaviors of adults partially mediated the behavior-related intervention effects on mental health and quality of life outcomes and pointed out the potential benefit of improving the overall pattern of physical activity and sleep on mental health [25]; another longitudinally observational study found out that sleep could be directly related to psychological health but could not mediate the relationship between PA and mental health [26]. However, so far, whether sleep has a moderator role between PA and mental health has not been very clear, and few studies took PA and sleep as a whole to explore their interactive regulation effects on mental health in longitudinal panel data.

Therefore, in this study, we surveyed the mental health regulated by the interactive effects of different PA levels, intensities, and sleep among college students in China. In detail, this study has three main purposes: (a) to provide longitudinal and relatively robust evidence on the interactive regulation effect of PA level and sleep health status on mental health; (b) to explore whether relationships between PA intensity and mental health are different among poor and good sleepers; (c) to determine the dose-response relationship between the optimal intensity PA and mental disorders. Moreover, we tried to collect and summarize biological evidence referring to previous studies which may support the interactive compensation effect of PA and sleep on mental health. We hypothesized that PA and sleep could affect mental health in an interactive way, and moderate intensity PA might be the optimal strategy to reduce negative emotions for poor sleepers. The present study may further provide integrative evidence on mental health and assist healthcare professionals in conducting appropriate PA interventions for those who suffer from sleep and mental problems.

## 2. Study Design and Methods

### 2.1. Design

This was a longitudinal study with four online measurements using structured questionnaires. Ethical approval was obtained from the Institutional Review Board of Tsinghua University (IRB: 20190091). Each measurement consisted of different intensity PA, PA level, sleep health status, and mental health indicators among a group of Chinese college students. Participants were instructed to complete the online questionnaires every half month. Four examinations were conducted in the daytime on 19 February 2020, 5 March 2020, 20 March 2020, and 6 April 2020.

### 2.2. Participants

Before the study, we posted recruitment information on the internet. Since we intended to explore the interactive effects of PA and sleep health on mental health via repeated measures, the inclusion criteria were: (1) college students with fluent reading ability, (2) non-disabled and physically healthy, (3) with a strong will to complete the whole process. A total of 66 qualified participants voluntarily participated in our research. Participants were informed of study details and asked to sign a consent form before starting the longitudinal survey. Also, they were told that they had the right to withdraw at any time. Participants were asked to complete and submit the online questionnaires on time at every turn. Since this was a longitudinal panel study, participants filling at least 2 questionnaires were considered valid. A total of 100, 70, and 30-yuan RMB (equivalent to 15, 10, and 4.5 US dollars, respectively) was offered to participants if they finished 4, 3, and 2 questionnaires, respectively.

### 2.3. Measures

#### 2.3.1. Physical Activity

Information on PA was collected by the 7-item short version of the International Physical Activity Questionnaire (IPAQ-S), which has been widely used in different countries for people aged between 15 and 69 years [27] and has been validated in the Chinese population with good reliability (Intraclass correlation coefficient, ICC = 0.79) [28]. The method for assessing PA has been clearly described in previous publications [26]. First, subjects were asked to classify typically weekly PA during the previous month into three intensity categories: light (e.g., walking), moderate (e.g., jogging), and vigorous (e.g., lifting) by reporting corresponding days per week and duration each time. An average metabolic equivalent value (MET) according to the Compendium of Physical Activities was assigned to each PA category: 3.3 for light, 4.0 for moderate, and 8.0 for vigorous [27,29]. Then, METs-minutes per week of light, moderate and vigorous intensity PA was calculated as the product of corresponding intensity (MET value) and total duration (min/week). Moreover, in accordance with the Guidelines for Data Processing and Analysis of the International Physical Activity Questionnaire (IPAQ), the physical activity of participants was divided into 4 incremental levels for further data analysis: inactive, low, moderate, and high.

#### 2.3.2. Sleep Status

Sleep was measured by Pittsburgh Sleep Quality Index (PSQI), which is commonly used to assess weekly general sleep disturbance in the last month [30]. We used the 19-item self-reported Chinese version of PSQI (i.e., C-PSQI), which has been validated in Chinese populations with good reliability (Cronbach α = 0.82) [31]. The C-PSQI consists of seven sleep components: subjective sleep quality, sleep latency, sleep duration, sleep efficiency, sleep disturbances, use of sleep medication, and daytime dysfunction. Each component was rated from 0 to 3. The total score was the sum of seven components, ranging from 0 to 21, with a higher score indicating greater sleep severity. According to previous studies, the total score less than or equal to 5 indicates healthy sleep status, while that greater than 5 mirrors some degrees of sleep disorders, i.e., unhealthy sleep status [31,32,33].

#### 2.3.3. Mental Health

Mental health was estimated by the 21-item Depression Anxiety Stress Scale (DASS-21) [34], which was extensively used to investigate recent negative emotions. It is widely used, and its reliability and validity have been proven in Chinese populations (Cronbach α = 0.74–0.84) [35,36]. The DASS-21 includes three components of stress, anxiety, and depression, each of which contains seven items. The method for assessing negative emotions has been clearly described in previous publications [26]. Each item was rated on a scale of 0–3, corresponding to “totally disagree”, “partially agree”, “mostly agree”, and “totally agree”. The score of stress, anxiety, and depression was calculated as the sum of seven related items’ scores, with values ranging from 0 to 21. The global DASS (G-DASS), as a general indicator of global negative emotions and mental distress, was calculated by adding scores of three components, ranging from 0 to 63. Additionally, higher scores of stress, anxiety, depression, and global DASS indicated more serious mental distress [34,35,37].

### 2.4. Statistical Analysis

A mixed-effect model with a random effect on individuals was adopted to first examine the effects of PA level, sleep status, and their interactions on mental health, and then assess the relationships between different intensity PA and mental health indicators mediated by healthy and unhealthy sleep status. Different intensity PA and mental health indicators were collected as continuous variables, while PA level was categorical variable and preprocessed into dummy variables for statistical analysis. The mixed-effect model can control time-invariant confounding variables and analyze unbalanced data [38]. In addition to the mixed-effect model, the dose-response relationship between the optimal intensity PA and mental health under different sleep statuses was explored by fitting a generalized additive model (GAM) with splines [39,40]. Data processing and statistical analysis were performed using R software, version 3.6.3 (R Project for Statistical Computing, Vienna, Austria). The mixed-effect model and GAM were modeled by using ‘lme4’ and ‘mgcv’ R-packages, respectively. A two-tailed *p* < 0.05 was considered statistically significant.

## 3. Results

### 3.1. Demographic Characteristics and Descriptive Analysis

Among all the follow-ups, 59 subjects completed four measurements, and 66 completed three of them. Therefore, the final valid number of participants was 66, and the total number of cases was 257. Table 1 shows the basic demographic information of the participants. Among 66 completers, 41 were female, and 25 were male, with a mean age around 21 years old. The majority of them were Han Chinese (92.42%) and lived in cities (77.27%). Furthermore, the mean and standard deviation of participants’ BMI was 21.11 ± 2.92 kg/m^2^, staying in the normal range, although males showed significantly higher BMI than females. No significant differences were observed in PA level, light PA, moderate PA, vigorous PA, PSQI score, and mental health indicators between four measurement time points. However, the number of participants with unhealthy sleep statuses was significantly higher at the second and third measurement time points.

### 3.2. Effects of Different PA Levels and Sleep Health Status on Mental Health

**Figure 1:**
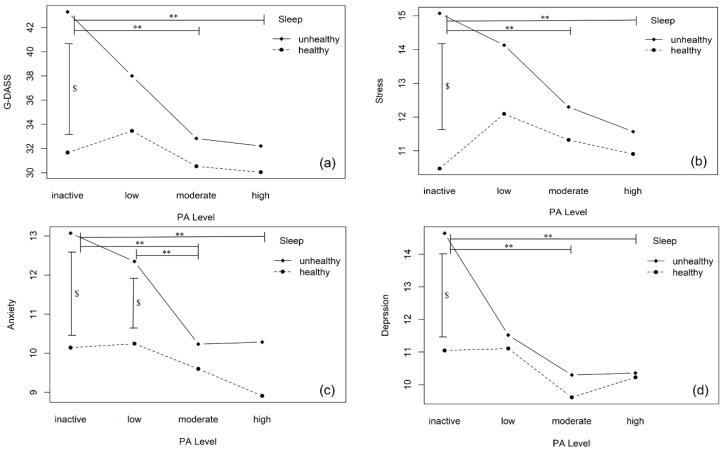
Effects of physical activity levels and sleep status on mental health. Note: PA, physical activity. G-DASS, global DASS, represents global negative emotions. The subgraph of (**a**–**d**) indicates the relationship between different PA levels and mental health indicators under healthy or unhealthy sleep status. Inactive, low, moderate, and high PA represent four incremental physical activity levels. Symbol ** indicates that there are significant differences at 0.05 level in mental health indicators among different levels of physical activity when sleep is at an unhealthy vs. healthy status. Symbol $ means there is a significant difference at 0.05 level observed in mental health indicators between healthy and unhealthy sleep under certain physical activity levels.

Figure 1 shows the two-way interaction plot of PA levels and sleep status on various mental health indicators. Sleep health status had a significantly main effect on G-DASS (*F* = 10.07, *p* = 0.002), stress (*F* = 11.33, *p* < 0.001), and anxiety (*F* = 10.64, *p* = 0.001); PA levels indicated a significant main effect on G-DASS (*F* = 3.46, *p* = 0.017), anxiety (*F* = 3.31, *p* = 0.021), and depression (*F* = 2.94, *p* = 0.034). Furthermore, their interactive effect was observed on G-DASS (*F* = 2.89, *p* = 0.036,) and stress (*F* = 3.50, *p* = 0.016). 

In addition, according to the Bonferroni correction applied results of simple effect analysis, when sleep was at a healthy status, no significant differences were observed on all mental health indicators at four PA levels (*p* > 0.05). However, when sleep was at an unhealthy status, scores of participants’ G-DASS (Mean_moderate-inactive_ [M_pa3-pa1_] = −7.182, *p* = 0.001; mean _high-inactive_ [M_pa4-pa1_] = −7.831, *p* = 0.004), stress (M_pa3-pa1_ = −3.011, *p* = 0.002; M_pa4-pa1_ = −3.237, *p* = 0.007), anxiety (M_pa3-pa1_ = −1.693, *p* = 0.019; M_pa4-pa1_ = −1.813, *p* = 0.039) and depression (M_pa3-pa1_ = −2.534, *p* = 0.003; M_pa4-pa1_ = −2.821, *p* = 0.007) were significantly lower in the PA-active group than the inactive group. Also, participants with moderate PA levels showed significantly less anxiety than those with low PA levels (*p* = 0.044). Similarly, when participants’ physical activity was at moderate or high levels, no significant difference was observed in mental health indicators at two sleep statuses, although poor sleepers showed elevated negative emotions. Nevertheless, when subjects were at inactive PA level, the G-DASS (Mean_unhealthy-healthy_ [M_h2-h1_] = 7.641, *p* < 0.001), stress (M_h2-h1_ = 3.706, *p* < 0.001), anxiety (M_h2-h1_ = 1.708, *p* < 0.020) and depression (M_h2-h1_ = 2.289, *p* = 0.008) significantly increased among poor sleepers. Still, poor sleepers showed significantly more anxiety than good sleepers under low PA levels (*p* < 0.036).

### 3.3. Relationships between Different Intensity PA and Mental Health under Varying Sleep Health Status

Table 2 shows the relationships between light, moderate, and vigorous intensity PA and different mental health indicators when individuals were at healthy and unhealthy sleep status. According to the results of the mixed-effect model, good sleepers did not reflect significant associations between different intensity PA and mental health (*p* > 0.05). However, when participants were at unhealthy sleep status, increasing moderate intensity PA was associated with decreased global negative emotions (*β* = −0.470, *p* = 0.011), stress (*β* = −0.184, *p* = 0.021), anxiety (*β* = −0.136, *p* = 0.033) and depression (*β* = −0.156, *p* = 0.012), indicating better mental health performance. Nevertheless, more light or vigorous-intensity PA was related to less negative emotions, from a descriptive perspective, among poor sleepers, but their relationships did not reach a significant level.

### 3.4. Dose-Response Relationship between Moderate Intensity PA and Mental Health under Varying Sleep Health Status

**Figure 2:**
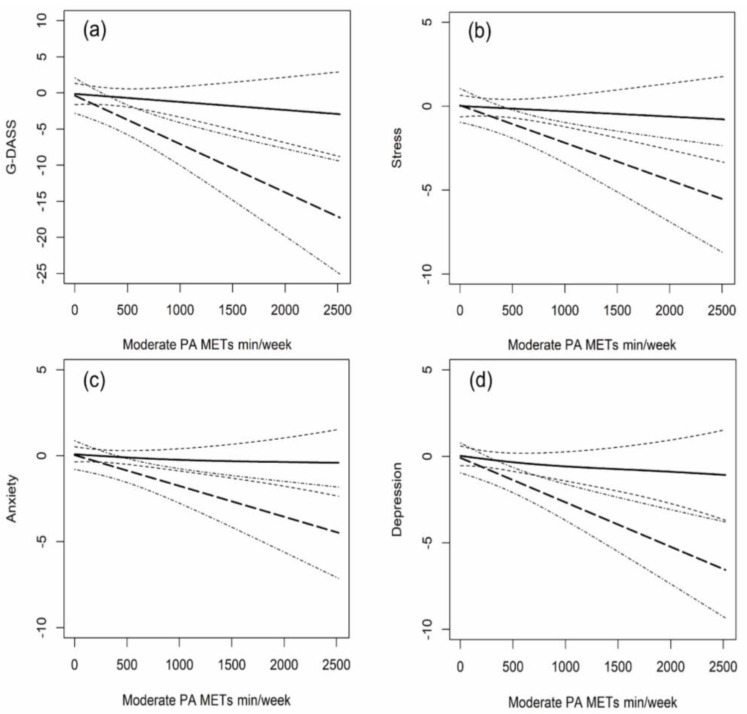
Dose-response relationships between moderate intensity physical activity and mental health indicators under healthy and unhealthy sleep status. Note: PA, physical activity. The subgraph of (**a**–**d**) indicates the dose-response relationship between moderate PA and different psychological indicators when at healthy or unhealthy sleep status. Non-linear splines for moderate intensity PA (METs-min/week) were used in a mixed-effect model with a random effect on individuals. The value of the *Y*-axis indicates the decrease of global negative emotions, stress, anxiety, and depression with the increasing moderate intensity PA. The bold-solid and bold-dash lines represent the relationship between moderate-intensity PA and mental health indicators under healthy and unhealthy sleep status, with thin-dash and thin-dot-dash lines indicating their 95% confidence interval, respectively. The range of stress, anxiety, and depression was all 0–21, and that of global negative emotions was 0–63.

Based on the analyses of Section 3.2 and Section 3.3, we further explored the dose-response relationships between moderate-intensity PA and mental health under healthy and unhealthy sleep statuses (Figure 2). The dose-response relationship between moderate-intensity PA and mental health indicators showed a downward near-linear trend among both good and poor sleepers, and no U-shaped or inverted U-shaped curve occurred. Furthermore, poor sleepers showed a much steeper downward dose-response curve than good sleepers between moderate-intensity PA and negative emotions, revealing a higher sensitivity. Also, increasing moderate intensity PA was slightly more effective in reducing depressive emotions among poor sleepers (Figure 2b–d).

## 4. Discussion

The focus of this longitudinal study is twofold: (1) to investigate the joint effect of PA and sleep on mental health; (2) to identify the most conducive PA pattern as a promising intervention strategy to improve sleep health and reduce negative emotions for poor sleepers. Our findings provide preliminarily longitudinal evidence that moderate-and-high level PA and healthy sleep status have an interactive compensation effect on reducing negative emotions caused by poor sleep health status or physical inactivity. Besides, for poor sleepers, the optimal compensation for alleviating negative emotions might be increasing the moderate intensity PA up to moderate-and-high PA levels.

In this longitudinal study, the phenomenon that both sleep and PA could play an important role in improving mental health is consistent with a large number of previous cross-sectional studies [9,18,41,42], indicating that regular PA and good sleep hygiene habits are associated with lower risk of psychological and psychiatric distress. Moreover, the interactive compensation mechanism found in the study was rarely mentioned in previous literature. Our study found that moderate-and-high level PA is helpful for poor sleepers to reduce mental impairment and good sleep status is beneficial for alleviating negative emotions caused by physical inactivity and sedentariness. However, unexpectedly, for good sleepers, increasing PA could not significantly promote mental health but only maintain it, which might be affected by the body’s stability and saturation mechanisms.

**Figure 3:**
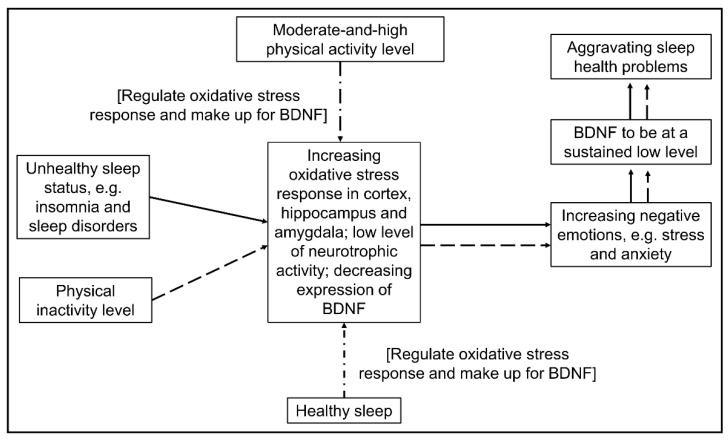
Possible schematic diagram of interactive compensation and regulation effects of physical activity and sleep on mental health referring to previous studies [43,44,45]. Note: BDNF, brain-derived neurotrophic factor. The solid line shows the influence path of sleep on mental health while the dashed line shows the influence path of physical activity on mental health. Furthermore, the dot-dash line represents the interactive regulation and compensation path.

Some biological evidence can support the above-mentioned interactive compensation mechanism (see Figure 3). Existing studies have reported that sleep problems such as sleep deprivation and sleep disorder can increase oxidative stress response in the prefrontal cortex, hippocampus, and amygdala, reduce cells in the prefrontal cortex and amygdala, and decrease the volume of the hippocampus, resulting in a low level of neurotrophic activity and reduced expression of brain-derived neurotrophic factor (BDNF), thus increasing negative emotions [43,44,45]. Furthermore, the BDNF level of individuals with severe stress, anxiety, or depression is prone to stay at a low level, which might lead to aggravating sleep health problems [43]. Interestingly, PA may break the vicious cycle because acute exercise and long-term regular exercise could help reduce oxidative stress products, enhance the activity of antioxidant enzymes, and increase the expression of BDNF, which effectively contribute to lessened mental distress [46,47]. Likewise, some biological evidence exists to support the offset effects of healthy sleep on people lacking physical activity [43,48]. However, good sleepers tend to present normal levels of oxidative stress responses, BDNF, and mental health. As a result, increased physical activity level may play a limited role to further improve mental health but maintain it. That is why the diminishing effect of PA on mental health demonstrated more effectively for poor sleepers.

We found that when sleep was at a healthy status, there was no significant relationship between light, moderate, and vigorous intensity PA and mental health indicators. In contrast, when sleep was at an unhealthy status, increasing moderate intensity PA was significantly associated with decreasing negative emotions, and the dose-response between them was near-linear. The finding could be interpreted by Figure 3 as well, i.e., the mental health of poor sleepers may be more vulnerable and sensitive and more likely to be improved by moderate intensity PA. Some previous studies also support our results [49,50], but others do not [6,51,52]. In fact, there is much inconsistency regarding the associations between PA intensity and mental health in existing cross-sectional research [6,15,49,50,51,52]. For example, some researchers found that only low- and moderate-intensity physical activity could reduce negative emotions and increase well-being [15], while others believe that high-intensity exercise has a better psychological promotion effect [52]. Also, there are many differences in evaluating the effects of PA intervention on mental health [7,53], which documented opposite results of the changing PA effects on mental and brain health. Three reasons may be considered: (a) previous studies could not control inter-person variation, which might lead to potential confounding variables and misleading results [14]; (b) previous intervention studies did not take both PA intensity and level into consideration; (c) previous studies ignored the interactive compensation effect between sleep and PA. However, this study has made some progress in these aspects. We used a mixed-effect model to control time-invariant confounding variables, such as age, gender, family background, and so on, and thus provide relatively robust results. Furthermore, we considered PA intensity, PA level, and sleep health status as an integrative behavioral system influencing mental health. Even so, considering that our sample size is not big enough, we cannot rule out that light or vigorous intensity PA might be more appropriate for other different groups of people with sleep and mental distress.

This study has several limitations. First, the sample size in our study was not that large, and Chinese college students were selected as a relatively unitary group as respondents. The results of this study population may not be representative of ordinary people of different ages, education levels, and countries. However, we collected longitudinal panel data to control confounding effects and observed significantly statistical results among variables. Therefore, the sample size in our study could provide robust longitudinal evidence to some extent. In addition, dose-response analysis between moderate-intensity PA and mental health indicators was to add spline curves to the independent variables, which required large number of samples to obtain a very accurate and stable relationship curve. However, limited to the sample size, although this study has done a simple dose-response analysis, we cannot rule out that the near-linear relationship curve may not be applicable to other groups. Hence, multivariate dose-response curves should be further analyzed after collecting enough data in the future. Moreover, we think that combined with a longitudinal panel study, objective PA and sleep measurement tools, such as accelerometers and polysomnography, are further needed for better exploring mental health research.

This study also has several strengths. First, this study conducted longitudinal panel surveys to assess the relationship between physical activity, sleep, and mental health. Unlike cross-sectional studies, this longitudinal study used a mixed-effect model with random effects on individuals to control inter-person variation and then provided much more robust and precise correlation evidence. Of note, to the best of our knowledge, this study is the first to investigate the interactive compensation effects of PA and sleep on mental health via longitudinal design. More importantly, we found that moderate-intensity PA might be the most beneficial tonic for people simultaneously suffering from sleep and mental health problems. Those findings may help broaden personalized and precise non-medicine intervention treatment for public health and integrate PA into sleep and mental health care.

## 5. Conclusions

In summary, this longitudinal panel study documents an interactive regulation and compensation effect of PA and sleep on mental health among Chinese college students, thus providing preliminary evidence that moderate-and-high level PA, among other factors, could attenuate the increasing mental distress for poor sleepers, and likewise, keeping a healthy sleep status could help compensate psychological well-being for those who are physically inactive. In addition, for those who are suffering from sleep and mental health problems (e.g., insomnia and depression), initiatively increasing moderate-intensity PA up to moderate-and-high PA levels is the most recommended approach to break the vicious circle. Under the background of COVID-19 sweeping across the world, our results highlight the relevance of good sleep hygiene for maintaining mental health and provide a promising public health intervention proposal for sleep and psychological sufferers by increasing moderate-intensity PA and raising PA levels.

## Figures and Tables

**Table 1:** Demographic information and descriptive analysis.

Variables	1st	2nd	3rd	4th	*p*
	N = 59	N = 66	N = 66	N = 66	
Categorical variables,					
N (%)					
Gender					
Males	18 (30.51)	25 (37.88)	25 (37.88)	25 (37.88)	0.78
Females	41 (69.49)	41 (62.12)	41 (62.12)	41 (62.12)	
Race					
Han	55 (93.22)	61 (92.42)	61 (92.42)	61 (92.42)	0.98
Minority	4 (6.78)	5 (7.58)	5 (7.58)	5 (7.58)	
Residence					
City	44 (74.58)	51 (77.27)	51 (77.27)	51 (77.27)	0.98
Countryside	15 (25.42)	15 (22.73)	15 (22.73)	15 (22.73)	
PA level					
Inactive	15 (25.42)	10 (15.15)	7 (10.61)	3 (4.55)	0.16
Low	14 (23.73)	19 (28.79)	22 (33.33)	21 (31.82)	
Moderate	23 (38.98)	29 (43.94)	27 (40.91)	31 (46.97)	
High	7 (11.87)	8 (12.12)	10 (15.15)	11 (16.66)	
Sleep status					
Healthy	50 (84.75)	38 (57.58)	34 (51.52)	54 (81.82)	<0.001
Unhealthy	9 (15.25)	28 (42.42)	32 (48.48)	12 (18.18)	
Continuous variables,					
Mean ± SD					
Age	20.46 ± 2.07	20.70 ± 2.10	20.70 ± 2.10	20.70 ± 2.10	0.56
BMI	21.0 ± 3.02	21.1 ± 2.92	21.1 ± 2.92	21.1 ± 2.92	0.81
Light PA	426.99 ± 553.11	327.50 ± 312.91	436.80 ± 516.49	505.25 ± 441.33	0.17
Moderate PA	360.34 ± 541.42	250.30 ± 374.19	383.94 ± 498.25	418.00 ± 538.27	0.24
Vigorous PA	280.68 ± 760.49	354.55 ± 613.40	294.55 ± 545.96	384.61 ± 849.67	0.53
PSQI	4.07 ± 2.00	5.74 ± 2.59	6.08 ± 1.99	3.76 ± 2.36	0.51
Stress	11.93 ± 3.08	12.32 ± 3.61	11.85 ± 3.86	11.74 ± 4.28	0.60
Anxiety	10.37 ± 2.71	10.41 ± 2.77	10.20 ± 2.81	10.09 ± 2.86	0.49
Depression	10.54 ± 3.19	10.59 ± 3.16	10.65 ± 3.12	10.83 ± 3.53	0.61
Global DASS	32.85 ± 7.82	33.42 ± 8.74	32.70 ± 9.04	32.67 ± 9.94	0.79

Note: N, the number of valid participants; SD, standard deviation; BMI, body mass index, calculated by the formula 10,000 × weight (kg)/height (cm)^2^; PA, physical activity, calculated as METs-minutes per week; PSQI, Pittsburgh Sleep Quality Index; DASS, a general indicator of the global negative emotions and mental disorders, was calculated by adding scores of stresses, anxiety, and depression. *p*-values are shown for differences between four measurement timepoints under the chi-square test (categorical variables) or a one-way ANOVA (continuous variables).

**Table 2:** Associations between various intensity physical activity and mental health indicators under healthy and unhealthy sleep status.

	Healthy Sleep Status		Unhealthy Sleep Status	
	*Β*	*p*	*β*	*p*
G-DASS				
100 × Light PA (METs-min/week)	0.055	0.619	−0.002	0.993
100 × Moderate PA (METs-min/week)	−0.153	0.185	**−0.470**	0.011 *
100 × Vigorous PA (METs-min/week)	−0.162	0.077	−0.134	0.299
Stress				
100 × Light PA (METs-min/week)	0.038	0.433	0.013	0.883
100 × Moderate PA (METs-min/week)	−0.070	0.166	**−0.184**	0.021 *
100 × Vigorous PA (METs-min/week)	−0.067	0.095	−0.062	0.248
Anxiety				
100 × Light PA (METs-min/week)	0.0001	0.998	0.032	0.654
100 × Moderate PA (METs-min/week)	−0.033	0.343	**−0.136**	0.033 *
100 × Vigorous PA (METs-min/week)	−0.039	0.165	−0.057	0.194
Depression				
100 × Light PA (METs-min/week)	0.010	0.814	−0.05	0.462
100 × Moderate PA (METs-min/week)	−0.049	0.276	**−0.156**	0.012 *
100 × Vigorous PA (METs-min/week)	−0.053	0.137	−0.019	0.683

Note: PA, physical activity; G-DASS, global DASS, represents global negative emotions. Light, moderate, and vigorous PA represent the different intensities of physical activity. * means a 0.05 significant level. *β*, point estimate, means the changing mean value of psychological indicators with the increase of 100 units of certain intensity PA. For instance, the value of −0.153 indicated when sleep was at a healthy status, and the global negative emotions would decrease by 0.153 with moderate PA increasing 100 METs-min/week. The ranges of stress, anxiety, and depression were all 0–21, and that of global negative emotions was 0–63. Statistically significant results at 0.05 level are presented in bold.

## Data Availability

The datasets generated and/or analyzed during the current study are not publicly available due to confidentially reasons but are available from the corresponding author upon reasonable request.

## Abbreviations

PA: Physical activity; BMI: Body mass index; MET: Metabolic equivalent; BDNF: Brain-derived neurotrophic factor; G-DASS: Global DASS, indicating scores of global negative emotions.
